# Efficacy of MMP-8 Level in Gingival Crevicular Fluid to Predict the Outcome of Nonsurgical Periodontal Treatment: A Systematic Review

**DOI:** 10.3390/ijerph19053131

**Published:** 2022-03-07

**Authors:** Sarhang Sarwat Gul, Faraedon Mostafa Zardawi, Ali Abbas Abdulkareem, Muhammad Saad Shaikh, Natheer Hashim Al-Rawi, Muhammad Sohail Zafar

**Affiliations:** 1 Department of Periodontics, College of Dentistry, University of Sulaimani, Sulaymaniyah 46001, Iraq; faraedon.mostafa@univsul.edu.iq; 2 Department of Periodontics, College of Dentistry, University of Baghdad, Baghdad 10011, Iraq; ali.abbas@codental.uobaghdad.edu.iq; 3 Department of Oral Biology, Sindh Institute of Oral Health Sciences, Jinnah Sindh Medical University, Karachi 75510, Pakistan; drsaadtanvir@gmail.com; 4 Department of Oral & Craniofacial Health Sciences, College of Dental Medicine, University of Sharjah, Sharjah 27272, United Arab Emirates; nhabdulla@sharjah.ac.ae; 5 Department of Restorative Dentistry, College of Dentistry, Taibah University, Al Madina, Al Munawwarra 41311, Saudi Arabia; mzafar@taibahu.edu.sa; 6 Department of Dental Materials, Islamic International Dental College, Riphah International University, Islamabad 44000, Pakistan

**Keywords:** matrix metalloproteinase-8, prediction, periodontitis, nonsurgical periodontal therapy, systematic review

## Abstract

Purpose: To explore whether baseline matrix metalloproteinase (MMP)-8 level in gingival crevicular fluid (GCF) (exposure) can predict the outcome (reduction in probing pocket depth (PPD) (outcome)) of nonsurgical periodontal therapy (NSPT) (manual or ultrasonic or both) in patients with periodontitis (population/problem) after 3 months. Methods: Six databases (PubMed, Cochrane library, ProQuest, Ovid, Scopus, EBSCO) were searched for relevant articles published until 30 July 2021. Retrieved articles were passed through a three-phase filtration process on the basis of the eligibility criteria. The primary outcome was the change in PPD after 3 months. Quality of the selected articles was assessed using Cochrane Risk of Bias tool (RoB2) and Risk of Bias In Non-Randomized Studies of Interventions (ROBINS-I) tools. Results: From 1306 articles, five were selected for analysis. The results showed high variations in the level of GCF MMP-8 level at baseline. The average amount of reduction in PPD was 1.20 and 2.30 mm for pockets with initial depth of 4–6 mm and >6 mm, respectively. Conclusion: On the basis of available evidence, it was not possible to reach a consensus on the ability of baseline GCF MMP-8 to forecast the outcome of NSPT. This could have been due to variation in clinical and laboratory techniques used. However, consistency in mean PPD reduction after 3 months was shown.

## 1. Introduction

Periodontal disease is a multifactorial inflammatory infection initiated as well as progressed by interactions between a dysbiotic biofilm and an aberrant immune response. As a result, supporting periodontal tissues will be permanently lost due to disruption of periodontal tissue homeostasis [1]. Diagnosis of periodontal diseases aims to identify the type, extent, and severity of the disease so that a suitable treatment plan and maintenance care can be implemented [2]. In contemporary clinical practice, the diagnosis mainly relies on clinical parameters, including bleeding upon probing (BOP), clinical attachment level (CAL), probing pocket depth (PPD), and alveolar bone loss [3,4]. 

Identifying the current state of the illness, future disease progression, and the success of periodontal therapy would be critical in developing a personalized treatment plan for each patient [5]. Although currently used techniques for diagnosing periodontal diseases, such as the conventional periodontal probing and radiograph, can precisely characterize the current state of the disease, they exhibit certain limitations [6] and cannot forecast the prognosis [7]. Gingivitis, which is the initial stage of periodontal disease, is a reversible condition, and has a favorable prognosis in the majority of patients [8]. However, this is not the case in periodontitis due to irreversible loss of periodontal tissues. According to the latest classification of periodontal diseases, clarity has been added to the previous classification through applying staging and grading systems for periodontitis [9]. The current severity and treatment complexity of periodontitis are represented by staging, whereas grading is used to assess the risk of disease progression and the expected response to periodontal therapy. However, there are some potential limitations of the grading system, leaving a space for biomarkers in saliva, gingival crevicular fluid (GCF), and serum to be added in the future to increase the degree of prognosis following periodontal therapy. Various biomarkers could represent potential surrogates but require further consideration in terms of their significance in the diagnoses, treatment, and degree of prognosis of periodontal diseases [9]. 

Until now, nonsurgical periodontal therapy (NSPT) has been used to remove supra- and subgingival biofilm and calculus as the first and most essential step for the treatment of periodontal disease [10]. However, even with the same baseline PPD, NSPT may not always yield desirable clinical improvements in all locations within the same patient over time [11,12]. This may be linked to the presence of high levels of key periodontal microbes including Aggregatibacter actinomycetemcomitans and Porphyromonas gingivalis in dental biofilm and immunological response of host tissues [13]. As a result, various options such as use of adjunct antimicrobials or surgical periodontal treatment are recommended [14,15]. Considering that the current method of diagnosis cannot predict the outcome of NSPT, the decision of using adjunct therapy or surgical intervention cannot be planned in the early stages. Due to close proximity to periodontal tissues and capability of detecting minute pathophysiological changes, GCF has demonstrated better reflectance of periodontal diseases compared to saliva and serum [16,17]. On the other hand, various molecular biomarkers of periodontitis in oral fluids have been examined to determine their appropriateness and effectiveness for predicting the outcome of NSPT, and significant progress has been achieved during the past decade [5,18]. One of the most studied biomarkers of periodontitis is matrix metalloproteinase (MMP)-8, a collagenase enzyme involved in the destruction of periodontal tissues and progression of periodontitis [19,20]. MMP-8 level is increased proportionally with the severity of periodontitis and could accurately reflect the progression and expected response to the treatment [19,20]. This latter notion has been supported by findings from previous studies [11,12]. 

Systematic reviews have shown MMP-8 to be a reliable tool for diagnosing periodontal diseases [21,22]. However, there is no systematic review determining the prognostic power of MMP-8 after NSPT. Thus, the purpose of the current systematic review was to explore the efficacy of MMP-8 in determining the outcome of NSPT with a follow-up period of at least 3 months.

## 2. Methods

### 2.1. Study Design and the Focused Question

This systematic review followed criteria stated in the updated Preferred Reporting Items for Systematic Reviews and Meta-Analyses (PRISMA) guidelines [23]. The research question for this review was formulated according to the Population, Exposure, Outcomes (PEO) criteria: “In patients with periodontitis treated with nonsurgical periodontal treatment (P) using either manual or ultrasonic instrumentation or both, is the baseline GCF MMP-8 level (E) efficient to predict the success/failure (reduction in PPD) (O) of treatment at three months follow-up?” The primary outcome was the change in mean PPD (mm) as assessed by a reduction of 1.26 mm (for pocket depth 4 to 6 mm) and 2.16 mm (for pocket depth ≥ 7 mm) after 3 months following NSPT [24]. The aforementioned amounts of reduction define successful NSPT after 3 months. 

### 2.2. Search Strategy and Eligibility Criteria

Using relevant Medical Subject Headings, relevant papers were retrieved from six databases (PubMed, Cochrane library, ProQuest, Ovid, Scopus, EBSCO) (MeSH). The search terms used were as follows: (“periodontal disease” OR “periodontitis”) AND (“gingival crevicular fluid” OR “gingival sulcular fluid”) AND (“periodontal therapy” OR “scaling and root planing” OR “subgingival debridement”) AND (“matrix metalloproteinases-8” OR “MMP-8” OR “biomarker”) AND (“prognostic” OR “prognosis” OR “point-of-care test”). A manual search was also conducted in relevant publications including the *Journal of Periodontology*, the *Journal of Clinical Periodontology*, the *Journal of Periodontal Research*, and the *International Journal of Dental Hygiene*. All studies published up to 30 July 2021 were included in the search. The following eligibility criteria were applied: 

Inclusion criteria

- Original studies (randomized and controlled clinical trials (RCT and CCT)) published in the English language.
- Monitoring periodontal treatment prognosis using concentration of whole MMP-8 (not activate MMP-8) at baseline.
- Subjects were healthy with no systemic illness.
- Non-smoker subjects suffering from periodontitis.
- Treated with NSPT (manual, ultrasonic, or both).
- Minimum follow-up duration of three months for clinical indicators and GCF MMP-8 level.
- One of the arms of the study to involve NSPT (manual or ultrasonic or both) only.
- No history of periodontal treatment in the past three months.

Exclusion criteria

- Case series, case reports, experimental or animal studies, observational studies, and review papers.
- Studies reporting pooled GCF MMP-8 of smokers and non-smokers as one group.
- Use of antibiotics or host modulation therapy (such as doxycycline) in the last three months.
- Pregnant or lactating women and patients on steroid or immunosuppressive therapy.
- Follow-up period of less than three months.

### 2.3. Literature Screening and Data Extraction

The retrieved articles went through a three-phase screening procedure based on the eligibility criteria after a preliminary search in the specified databases. This began with a title screening, the second step included an abstract screening, and the third phase included a thorough full-text reading.

Screening was performed separately by two authors (N.H.A. and A.A.A.), and any discrepancies were addressed by discussion with a third reviewer (S.S.G.). Cohen’s kappa was used to measure the level of inter-reviewer agreement [25]. Published articles that met the qualifying criteria were included. Data including the author’s name/year, the study’s design, sample characteristics, interventions, follow-up periods, and procedures for collecting GCF and assaying MMP-8 levels were retrieved. In addition, sample elution/storage techniques, MMP-8 concentration in GCF expressed in ng/mL, age characteristics, PPD measurements in detail, and variations in PPD after 3 months of NSPT were evaluated. The results were based on groups who met the eligibility criteria and were solely given NSPT without any additional local or systemic adjunct(s).

### 2.4. Quality Appraisal

Two researchers (M.S.S. and M.S.Z.) performed the quality appraisal of the papers included. For RCT, the quality of the included studies was evaluated via the Cochrane Risk of Bias tool (RoB2) [26] and the Risk of Bias In Non-Randomized Studies of Interventions (ROBINS-I) tool for CCT [27]. A total of five domains were examined for the RoB2 test, with judgments ranging from minimal risk of bias to some concerns to high bias risk. Overall, the risk of bias usually parallels to the worst bias risk in any of the domains. Nevertheless, if a publication is rated as having “some concerns” about risk of bias in several domains, it may be classed as high risk of bias overall. 

The ROBINS-I instrument assesses a total of seven domains, with low, moderate, severe, and critical risk of bias being the judgments. The low risk of ROBINS-I indicates a high-quality clinical study. Overall, the article is considered to be at low bias risk for each domain; for moderate risk, the paper is classified to be at low/moderate bias risk for every domain; for serious risk, the study is categorized to be at serious bias risk in at least one domain, but not at critical bias risk in any domain; and for critical risk, the article is classified to be at critical bias risk in at least one domain.

## 3. Results

### 3.1. Selection of Studies

**Figure 1:**
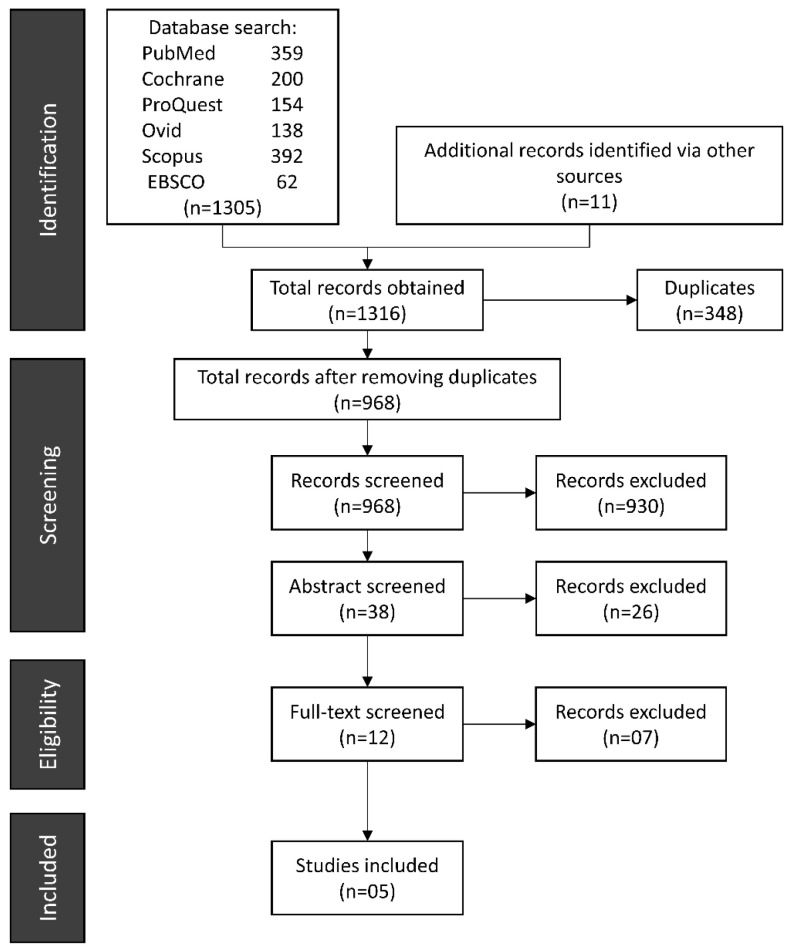
PRISMA flowchart.

A total of 1306 items were found during the search process. After removing duplicates, 968 articles remained. Two reviewers used the eligibility criteria to determine whether the articles should be included or excluded on the basis of their titles. This step eliminated 930 publications, followed by the exclusion of another 26 papers on the basis of abstract screening. For full-text reading, 12 articles were nominated (Figure 1). Finally, five articles [28,29,30,31,32] fulfilling the eligibility criteria were further analyzed for data extraction and answering the PEO question. The study periods of included papers were from 2006–2021. Table S1 describes the reasons for exclusion after reading the abstract. After full-text reading, another seven articles [12,33,34,35,36,37,38] were excluded (Table 1). The computed Cohen’s kappa values for inter-reviewer agreement for the first, second, and third phases of the screening procedure were 0.77, 0.83, and 0.89, respectively, indicating considerable to almost perfect agreement between the two reviewers.

### 3.2. Study Design and Populations

Four (out of five) studies followed the parallel-arm design [28,29,31,32], whereas only one followed the split-mouth design [30]. Three papers were RCTs [30,31,32], while the other two were CCTs [28,29] (Table 2). For groups included in the final analysis that received NSPT only, the lowest sample size was five [28] and the largest was 29 [31]. In any one study, the widest age range reported was from 18 years to 70 years [30]. Meanwhile, the minimum mean age was 28.9 years [32] and the maximum was 46 years [30] (Table 3). Conversely, in another study [28], no details regarding age were mentioned (Table 3).

### 3.3. Follow-Up Periods 

In three studies, the minimal follow-up time was three months [29,30,31]. The longest follow-up period was 12 months, which was only seen in one study [28]. Finally, the most recent study demonstrated a 6 month follow-up period following NSPT [32] (Table 2).

### 3.4. GCF Collection, Elution, and Storage Methods 

In all included papers, GCF was collected by inserting an absorbent paper into the sulcus/pocket and leaving it for 30 s. Only two studies [29,31] used the same paper strip brand (PerioPaper: Oraflow, Inc., NY, Simthtown, NY, USA). Conversely, Pourabbas et al. (2014) [30] utilized a different brand of paper from the same company (PerioCol paper, Oraflow, NY, Simthtown, NY, USA). One study did not specify the type and manufacturer of the paper strip used [28], while the remaining study [32] collected GCF using prefabricated paper points (Table 2). 

Phosphate-buffered saline (PBS) was used in three trials to elute GCF from paper strips; two of them [29,32] used 1 mL PBS, while the third one [30] used 250 µL PBS. The GCF samples in one study [28] were eluted using different solutions. Erbil et al. (2020) did not provide any details on the elution solution or duration. Briefly, the duration and technique of elution differed amongst the trials, with the longest elution duration being 2 h [30], followed by 40 min [29], 15 min [32], and 5 min [28]. Furthermore, eluted GCF samples in one study [29] were further centrifuged. In two studies, samples were kept at −70 °C [29,30]. Conversely, the samples were kept at −80 °C by Erbil et al. (2020) and at −20 °C by Taalab et al. (2021). However, no storage was used in one particular study [28]; instead, the samples were directly analyzed. None of the other studies provided information on how long the samples were kept before being analyzed (Table 2).

### 3.5. Biochemical Assays and Concentration of GCF MMP-8

Three studies [30,31,32] used enzyme-linked immunosorbent assay (ELISA) for MMP-8 analysis. One study [29] used the multiplex bead method, and the other study [28] used a MMP-8-specific periodontal chair side dipstick test and time-resolved immunofluorometric assay (TR-IFMA) to measure GCF MMP-8 levels (Table 2). The highest concentration (mean ± SD) of GCF MMP-8 measured at baseline was 3997 ± 3126 ng/mL [28], and the lowest concentration was 2.00 ± 1.60 ng/mL [32]. Two studies reported close values of GCF MMP-8 concentration of 306.34 ± 255.97 ng/mL [30] and 331.50 ± 299.70 ng/mL [31]. Conversely, the results of Correa et al. (2008) demonstrated concentration as a median and interquartile range of 20.6 (11.5/32.3) ng/mL (Table 2).

### 3.6. Measurement of PPD and Case Definition of Periodontitis

Three studies [29,30,31] used six sites per tooth to assess PPD, one study did not report how many sites were used to measure PPD [32], and Mäntylä et al. (2006) [28] did not provide any information on measuring clinical parameters (Table 2). Four studies used standard manual probes, namely, UNC-15 [30], North Carolina probe [29], and Williams periodontal probe [31,32] (Table 3).

Two studies [29,30] used a combination of CAL and PPD to define periodontitis patients. Confirmation of alveolar bone loss using radiographs was observed in two studies [28,32]. Only one study [31] relied on presence of periodontal pockets (PPD ≥ 5 mm) in at least four teeth to define periodontitis. All the studies defined the minimum number of natural teeth present at the time of recruitment, except for one study [32], which excluded those who lost their teeth due to periodontitis purely in accordance with the latest classification of periodontal and peri-implant diseases and conditions [3] (Table 3).

### 3.7. Changes in PPD 

The PPD ranged between 4 and 6 mm at baseline in four studies, with PPD (mean ± SD) reported as 4.47 ± 1.23 mm [30], 5.00 ± 2.10 mm [28], 4.70 ± 0.70 mm [31], and 5.50 ± 1.10 mm [32]. The amount of the reductions in mean PPD (∆ mean PPD) at first visit following NSPT were 1.27 ± 0.08 mm, 2.20 ± 0.80 mm, 1.40 ± 0.01 mm, and 1.20 ± 0.40 mm, respectively. In addition, Erbil et al. [31] reported mean PPD > 6 mm at baseline (7.50 ± 0.70 mm) separately, which showed a reduction of 2.30 ± 0.50 mm after 3 months. Correa et al. (2008) [29] reported a median value of 3.60 mm for PPD, and this was reduced by 1.20 mm after 3 months. Three studies [28] showed successful PPD reduction after 3 months of NSPT, as previously defined [24], while results of two studies [29,32] failed to report similar outcomes (Table 3).

### 3.8. Quality Assessment

**Figure 2:**
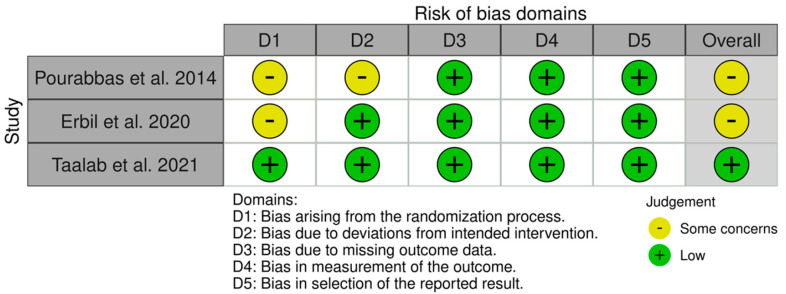
Individual domain results for studies assessed by RoB2 tool.

Three studies (RCT) were evaluated using the RoB2 tool, and two studies (CCT) were evaluated using the ROBINS-I tool. Two studies [30,31] were assessed as having some concerns, mostly due to issues with the randomization procedure, whereas one study [32] was categorized as having a minimal bias risk. Overall, the bias risk was categorized as some concerns by the RoB2 tool (Figure 2).

**Figure 3:**
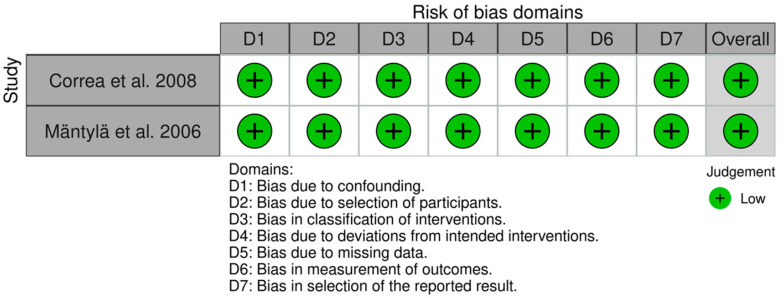
Individual domain results for studies assessed by ROBINS-I tool.

The two CCT studies [28,29] were classed as low bias risk using the ROBINS-I method, with an overall bias risk rating of low (Figure 3).

## 4. Discussion

The present study systematically reviewed clinical research reporting the efficacy of MMP-8 in forecasting the outcome of NSPT. Generally, periodontitis is initially treated with NSPT coupled with strict maintenance by oral hygiene measures. Periodontal pockets that do not respond to mechanical debridement may require surgical intervention [39]. Therefore, the treatment plan of periodontitis may be a complicated process affected by individual variations. Even seemingly treatable cases may require more sophisticated treatments [6,40]. Predicting the outcomes of NSPT at baseline may have significant benefits of time- and cost-effectiveness. Traditional techniques such as periodontal probing and radiographs provide necessary information to make a diagnosis; however, they provide limited information on prognosis following NSPT [6,40]. The MMP-8 biomarker found in the subgingival region demonstrated optimistic results in the diagnosis and may be useful for predicting site-specific prognosis following NSPT. Therefore, this review was designed to answer a focused question regarding whether GCF MMP-8 level at baseline can be used to predict site-specific outcome of NSPT (manual, ultrasonic, or both) in patients with periodontitis. 

The main finding of the current review was a high variation in the level of GCF MMP-8 as measured at baseline (primary outcome) among selected studies. The reported levels ranged from as low as 2.00 ± 1.60 ng/mL [32] to 3997 ± 3126 ng/mL [28]. These high differences in MMP-8 level could be associated with methodological variations in both the clinical and laboratory techniques used, as described below. For example, the selected studies showed variations in the sites selected for collecting GCF. Three studies [30,31,32] selected the deepest pocket for sampling GCF. Correa et al. (2008) determined the number of pockets, PPD and CAL as eligibility criteria for the sample, while Mäntylä et al. (2006) did not report any details about the sites included for GCF collection. In addition, only single-rooted teeth were selected in one paper [31]. 

Assays used to determine MMP-8 concentration also varied, with ELISA being the most common method [30,31,32], while others used chair side dipstick test/IFMA [28] and multiplex bead technique [29]. ELISA is one of the most widely used and cost-effective antigen detection methods worldwide. However, because of cross reactivity, sensitivity to background, and errors in executing washing procedures, ELISA has the potential to provide false-negative or -positive findings [41]. Additionally, signal stability is another issue with ELISA, and the numerous washing procedures may cause a weak antibody–protein interaction that may not be detected [41]. Accuracy, sensitivity, and specificity of detecting proteins are increased when TR-IFMA is used compared to the ELISA [42]. Eliminating the interference from optical background and greater concentration of highly specific antibodies that minimize cross-reactivity are the main reasons for confirming the validity of TR-IFMA results [43]. Multiplex bead array assays are ELISA-based immunoassays that allow for the simultaneous detection of several antigens. However, the chances of cross-reactivity between various analytes in the sample are enhanced, the latter point potentially being a source of errors [44].

For collecting GCF samples, all investigations used the same methodology (Brill’s technique), which involved inserting a paper strip until mild resistance was felt and leaving it in place for half a minute. However, differences in the brand of paper strip and its manufacturer’s origin were observed, which may have affected the quantity of the collected sample. Two studies [29,31] used PerioPaper (Oraflow, Inc., Smithtown, NY, USA), which is designated by the company for collecting GCF samples (1.20 µL) from healthy and gingivitis sites, while PerioCol paper is a type indicated for periodontitis cases and was used by only one study [30]. The latter type of paper strips is longer than the former and allows for the collection of a larger volume of GCF (2.00 µL). Prefabricated paper points were used by another study [32] and were not validated for their efficacy in collecting GCF. 

After collecting GCF, paper strips were placed in PBS for eluting components of GCF [29,30,32]. The studies showed variations in the time elapsed for elution and the volume used, which ultimately may have affected the dilution of the sample and retrieved amount of MMP-8 from the paper strips. In addition, when PBS is frozen, formation of phosphoric acid is possible, which negatively affects the constituents, i.e., proteins and their functionality [45,46]. Therefore, when considering PBS for freezing, altering the concentration of NaCl, one of the PBS constituents, is recommended [47]. In contrast to PBS, the pH of N-2-hydroxyethylpiperazine-N-2′-ethanesulfonic acid (HEPES) buffer, which was used by only one study for cryopreservation [28], tends to increase, i.e., becoming alkaline in pH upon freezing [48]. Interestingly, using a mixture of phosphate-buffered solution/HEPES (1:1 ratio) tends to produce a stable pH when frozen [48]. 

Another point to consider is whether GCF MMP-8 in the samples was directly assayed after collection, which was observed in one study [28], or frozen-thawed and then measured, as was performed by the other four studies [29,30,31,32]. Despite the fact that MMP-8 was found to be stable after multiple freeze–thaw cycles in a prior study [49], repetitive freezing and thawing causes denaturation of proteins that would otherwise be exposed to low pH and high buffer salts [46]. 

When attempting to test MMP-8 levels over a prolonged period of time, another issue to consider is storage temperature. Although biological fluids can be suitably frozen at temperatures ranging from −20 °C to −80 °C, when the stability of samples was practically approximated using the Arrhenius equation, the findings revealed that around 90% of the original protein content could be preserved over a period of months at −20 °C and for several decades at −75 °C [50]. This is in line with findings from this review, which found that one of the trials [32] maintained the sample at −20 °C and reported the lowest MMP-8 content when compared to other studies that froze the samples at temperatures ranging from −70 °C to −80 °C. 

The mean amount of reduction in PPD was ≥1.20 mm, for pockets with initial depth of 4 to 6 mm, after 3 months. Additionally, mean reduction in PPD of > 6 mm after the same period was 2.30 mm. These results were consistent with changes in PPD reported in a previous study [24]. Overall, the amount of reduction in the depth of periodontal pockets among the reviewed studies was not consistent with the level of GCF MMP-8 at baseline. Only two studies [30,31] found extremely similar baseline levels of MMP-8, with mean PPD reductions ranging from 1.27 [30] to 1.40 mm [31]. As a result, it was not possible to perform a quantitative analysis in this review. More research is needed to establish a precise cut-off value for MMP-8 that may be utilized to predict NSPT results, considering rigorous uniformity for GCF collection, elution, types/volumes of solutions, measurement procedures, and storage. Furthermore, due to the complex nature of periodontal disease, an individual biomarker might not be enough to reflect the overall changes in periodontal tissue. Therefore, using two or more biomarkers to diagnose/monitor periodontal disease might be more advantageous. In addition, a single biomarker might be easily influenced by both systemic and local factors. 

Briefly, limitations of the current review can be attributed to the inclusion of systematically healthy and nonsmoker subjects. These factors could alter the expression of a wide range of biomarkers including MMP-8. In addition, the main aim was to estimate a site-specific baseline level of GCF MMP-8 to predict the outcome of NSPT. However, raw data were not available, and the interpretation was based on the pooled mean of MMP-8 level and clinical parameters, which further limited determination of the predictive value of this biomarker. Despite these limitations, as far as we are aware, this study is one of the few qualitative studies aimed at identifying a site-specific MMP-8 level as a predictive tool.

This systematic review showed high variations in GCF MMP-8 levels at baseline due to the different clinical and biochemical analyses used by the studies. Standardization of the aforementioned parameters is required to reach a consensus about a valid cut-off value for this biomarker if intended to be used as a prognostic tool. Indeed, introduction of diagnostic/prognostic biomarker-based chairside technique in dental practice is recommended. These revolutionary tools would be time-saving and minimize human errors, thereby aiding in outlining tailored treatment plans and improving outcomes. However, cost-effectiveness value of using diagnostic biomarkers should be crucially estimated to avoid overburdening the patients and healthcare systems with unreasonably extra expenses.

## 5. Conclusions

On the basis of analyses from this systematic review, the available evidence did not permit the reaching of a consensus on a reliable cut-off value for baseline GCF MMP-8 to anticipate site-specific outcomes of NSPT. This indicates laboratory techniques need further standardizations to allow for comparison between studies and to draw firm conclusions.

## Figures and Tables

**Table 1:** Reasons for exclusion after full-text evaluation.

	Author, Year	Reason(s) for Exclusion
1	Eltas, Orbak [33]	Smoking status was not determined
2	Azmak, Atilla, Luoto, Sorsa [34]	
3	Gul, Griffiths, Stafford, Al-Zubidi, Rawlinson, Douglas [12]	Data of smoker and non-smoker individuals were combined
4	Emingil, Han, Gürkan, Berdeli, Tervahartiala, Salo, Pussinen, Köse, Atilla, Sorsa [35]	
5	Cosgarea, Eick, Jepsen, Arweiler, Juncar, Tristiu, Salvi, Heumann, Sculean [34]	
6	Emingil, Han, Ozdemir, Tervahartiala, Vural, Atilla, Baylas, Sorsa [37]	
7	Marcaccini, Meschiari, Zuardi, de Sousa, Taba, Teofilo, Jacob-Ferreira, Tanus-Santos, Novaes, Gerlach [38]	Concentration of whole MMP-8 was not reported

MMP-8: matrix metalloproteinase-8.

**Table 2:** Baseline concentration of GCF MMP-8 (primary outcome).

Author, Year	Study Design	Sample Characteristics	Interventions/Follow-Up for GCF Collection	GCF Collection Method/MMP-8 Assays	Sample Elution/Storage	Baseline GCF MMP-8 (ng/mL) ^ǂ^
Mäntylä et al., 2006 [28]	Prospective clinical trial with parallel-arm design	Periodontitis, patients Non-smoker (*n* = 5), smoker (*n* = 11)	Oral hygiene instructions and SD ^Ɨ^ GCF samples were collected bimonthly over 12 months	Filter paper ^#^ was placed into the gingival sulcus for 30 s Selected sites/teeth: NR MMP-8-specific periodontal chair side dipstick test and time-resolved IFMA	The strip was put in a test tube containing 0.5 mL of pH 7.4 HEPES-buffer. The proteins absorbed by the sample strip were then eluted for 5 min in the buffer. There was no storage used.	3997 ± 3126 ^¶^
Correa et al., 2008 [29]	Clinical trial with parallel-arm design	Control arm: systemically healthy with periodontitis (*n* = 26) Study: patients with type 2 diabetes mellitus and periodontitis (*n* = 23)	Oral hygiene instructions and supra- and SD via manual instruments ^Ɨ^ Samples from GCF were taken at baseline and after 3 months	GCF was collected from five or six deep sites (PD ≥ 5 mm, CAL ≥ 4 mm, and BOP) and five or six shallow sites (PD ≤ 3 mm, CAL ≤ 2 mm, and BOP) in separate non-adjacent teeth GCF was collected for 30 s with paper strips (PerioPaper: Oraflow, Inc., NY, USA) placed in sulcus until resistance was felt Concentrations were determined using an antibody pair and recombinant MMP-8, and the results were evaluated using a multiplex bead method	Each subject’s samples from the same site type (deep or shallow) were combined together in an Eppendorf tube containing 1 mL PBS. After 40 min of elution at room temperature, the samples were centrifuged for 10 min at 3000× *g*, and the supernatant was collected and promptly frozen at −70 °C.	20.6 (11.5/32.3) ^§^
Pourabbas et al., 2014 [30]	Split-mouth RCT	Chronic periodontitis patients (*n* = 22) One side assigned as a control and the other as a study side	SD only ^Ɨ^ GCF collection was done at the start of the study and 3 months afterwards	Sterile paper strips (PerioCol paper, Oraflow, NY, USA) were placed into the deepest portion of the periodontal pockets of all treated teeth and kept in place for 30 s The sandwich ELISA was used for GCF MMP-8 analysis	The strips were placed in sterile microtubes containing 250 µL PBS. The samples were kept at −70 °C after being left at 4 °C for up to 2 h.	306.34 ± 255.97 ^¶^
Erbil et al., 2020 [31]	Multi-center, parallel RCT	Patients with periodontitis Control (*n* = 29), study (*n* = 30)	SD only ^Ɨ^ The data were recorded at baseline, 6 weeks, and 3 months following the therapy	For sampling, the deepest pockets (maximum of 9 mm) of single-rooted teeth were chosen (4 sites/patient); for 30 s, paper strips (PerioPaper: Oraflow, Inc., NY, USA) were carefully placed into the pockets GCF MMP-8 concentration was determined by ELISA	Each patient’s pooled strips were put in sterile Eppendorf tubes and kept at −80 °C.	331.50 ± 299.70 ^¶^
Taalab et al., 2021 [32]	Parallel arm RCT	Patients with stage 2, grade B periodontitis Patients (*n* = 30) were randomly and equally assigned to study or control group	Full mouth supra- and SD with manual and ultrasonic scalers, as well as advice about oral hygiene measures ^Ɨ^ GCF was collected at 1, 3, and 6 months following therapy	GCF samples were obtained by placing prefabricated paper points into the deepest location until resistance was felt, then left for 30 s Sandwich ELISA was used to assay level of GCF MMP-8	The samples were diluted in 1 mL of PBS. After leaving samples for 15 min in PBS, they were frozen at −20 °C.	2.00 ± 1.60 ^¶^

AZM: azithromycin, NSPT: nonsurgical periodontal therapy, MMP-8: matrix metalloproteinase-8, TIMP-1: tissue inhibitor of MMPs, MPO: myeloperoxidase, GCF: gingival crevicular fluid, PDT: photodynamic therapy, PPD: probing pocket depth, RCT: randomized clinical trial, ELISA: enzyme-linked immunosorbent assay, IFMA: immunofluorometric assay, CHX: chlorhexidine, IL: interleukin, AMX: amoxicillin, MET: metronidazole, SD: subgingival debridement, Er, Cr:YSGG: erbium, chromium:yttrium–scandium–gallium–garnet, SBI: sulcus bleeding index, PBS: phosphate-buffered saline, NR: not reported, HEPES: N-2-hydroxyethylpiperazine-N-2′-ethanesulfonic acid. ^Ɨ^ Intervention for control group only. ^ǂ^ Results at baseline for individuals who received subgingival debridement only without any other local or systemic adjunct(s). ^#^ Brand not reported. ^¶^ Mean ± SD. ^§^ Median and IQ range.

**Table 3:** Change in probing pocket depth (secondary outcomes) in response to nonsurgical periodontal therapy.

Author, Year	Age Range Mean ± SD (Years)	Case Definition of Periodontitis	Details of PPD Measurement	Baseline PPD (mm)	∆ Mean PPD Reduction (mm) ^ǂ^
Mäntylä et al., 2006 [28]	NR NR	At least 20 teeth, and at least five locations with 4 mm PPD and radiographic bone loss	Details of measurements: NR Clinical parameters were measured by manual periodontal probe (Type: NR)	5.00 ± 2.10 ^¶^	2.20 ± 0.80 ^¶^ *
Correa et al., 2008 [29]	NR 41.60 ± 7.10	≥15 teeth, at least five teeth with one or more sites with PPD ≥ 5 mm, CAL ≥ 4 mm, visible plaque, and BOP	PPD was measured at six sites per tooth using a conventional manual probe (North Carolina probe)	3.60 ^§^	1.20 ^§^
Pourabbas et al., 2014 [30]	18 to 70 46 ± 8	≥12 natural teeth, with at least three in each quadrant; ≥3 mm CAL in at least 30% of the teeth; and ≥1 site/quadrant with PPD ≥ 4 mm and BOP	Using a conventional manual probe (UNC-15), PPD was measured at six sites per tooth	4.47 ± 1.23 ^¶^	1.27 ± 0.08 ^¶^ *
Erbil et al., 2020 [31]	31 to 56 39.72 ± 6.16	More than three teeth in each quadrant; at least four periodontal pockets with a PPD ≥ 5 mm	PPD was measured at six sites per tooth by a standard manual probe (Williams periodontal probe)	4–6 mm: 4.70 ± 0.70 ^¶^ >6 mm: 7.50 ± 0.70 ^¶^	4–6 mm: 1.40 ± 0.01 ^¶^ * >6 mm: 2.30 ± 0.50 ^¶^ *
Taalab et al., 2021 [32]	25–50 28.9 ± 6.30	CAL = 3 to 4 mm, BOP, and radiographic horizontal bone loss in the root’s coronal third (15–33%); no tooth loss as a result of periodontitis	PPD was measured by a standard manual probe (Williams periodontal probe)	5.50 ± 1.10 ^¶^	1.20 ± 0.40 ^¶^

NR: not reported, PI: plaque index, BOP: bleeding on probing, PPD: probing pocket depth, CAL: clinical attachment loss, CEJ: cemento-enamel junction, GR: gingival recession, PBI: papilla bleeding index, VPI: visible plaque index, GBI: gingival bleeding index, NSPT: nonsurgical periodontal therapy, SBI: sulcus bleeding index. ^ǂ^ Mean reduction in PPD at first visit 3 months after finishing NSPT for groups who received subgingival debridement only without any other local or systemic adjunct(s). ^¶^ Mean ± SD. ^§^ Median value. * Successful reduction of PPD after 3 months of NSPT.

## Data Availability

The data presented in this study are available on request from the corresponding author.

## Supplementary Materials

The following supporting information can be downloaded at https://www.mdpi.com/article/10.3390/ijerph19053131/s1; Supplementary Table S1: Reasons for exclusion after abstract review. References [20,51,52,53,54,55,56,57,58,59,60,61,62,63,64,65,66,67,68,69,70,71,72,73,74,75] have been cited in the Supplementary Materials.
