# Supplementary material for: Efficacy of MMP-8 Level in Gingival Crevicular Fluid to Predict the Outcome of Nonsurgical Periodontal Treatment: A Systematic Review
Source: Int J Environ Res Public Health. 2022 Mar 7;19(5):3131. doi: 10.3390/ijerph19053131 (PMC8910039; doi:10.3390/ijerph19053131)
Supplement: Supplementary file 1 [file ijerph-19-03131-s001.zip › ijerph-1561950-supplementary.pdf]

Table S1: Reasons for exclusion after abstract review.

| | Author, year | Reason(s) for exclusion |
|---|---|---|
| 1 | Kinane et al. (2003)[51] | Smoking status not determined |
| 2 | Kinney et al. (2014) [52] | |
| 3 | Choi et al. (2004) [53] | |
| 4 | Skurska et al. (2015) [54] | Inclusion of adjunctive treatment in all groups |
| 5 | Nardi et al. (2020) [55] | Salivary MMP-8 |
| 6 | Liu et al. (2020) [56] | |
| 7 | Buduneli et al. (2002) [57] | Follow-up less than 3-months |
| 8 | Farhad et al. (2013) [58] | |
| 9 | Kurtis et al. (2007) [59] | |
| 10 | Chen et al. (2000) [60] | |
| 11 | Ozgören et al. (2014) [61] | |
| 12 | Tüter et al. (2010) [62] | |
| 13 | Jentsch et al. (2016) [63] | Antibiotics were used |
| 14 | Gonçalves et al. (2013) [64] | Inclusion of children and young adults |
| 15 | Leppilahti et al. (2015) [65] | Summary of several RCT |
| 16 | Sorsa et al. (2016) [66] | Narrative review |
| 17 | de Oliveira et al. (2009) [67] | MMP-8 was not included |
| 18 | Holmlund et al. (2004) [68] | |
| 19 | Emingil et al. (2004) [69] | |
| 20 | Reddy et al. (2012) [70] | |
| 21 | Koromantzos et al. (2012) [71] | Study on diabetic patients |
| 22 | Okuda et al. (2001) [72] | Surgical intervention |
| 23 | Górska and Nedzi-Góra (2006) [73] | GCF was not collected |
| 24 | Ağan et al. (2006) [74] | Doxycycline applied in all groups |
| 25 | Kurgan et al. (2016) [75] | NSPT for patients with rheumatoid arthritis |
| 26 | Leppilahti et al. (2014) [76] | 3-month's results not reported |

MMP-8: matrix metalloproteinase, RCT: randomized clinical trial, GCF: gingival crevicular fluid, NSPT: nonsurgical periodontal therapy

References

51. KINANE, D. F., DARBY, I. B., SAID, S., LUOTO, H., SORSA, T., TIKANOJA, S. & MÄNTYLÄ, P. 2003. Changes in gingival crevicular fluid matrix metalloproteinase-8 levels during periodontal treatment and maintenance. *J Periodontal Res,* 38**,** 400-4.

52. KINNEY, J. S., MORELLI, T., OH, M., BRAUN, T. M., RAMSEIER, C. A., SUGAI, J. V. & GIANNOBILE, W. V. 2014. Crevicular fluid biomarkers and periodontal disease progression. *J Clin Periodontol,* 41**,** 113-120.
53. CHOI, D. H., MOON, I. S., CHOI, B. K., PAIK, J. W., KIM, Y. S., CHOI, S. H. & KIM, C. K. 2004. Effects of sub-antimicrobial dose doxycycline therapy on crevicular fluid MMP-8, and gingival tissue MMP-9, TIMP-1 and IL-6 levels in chronic periodontitis. *J Periodontal Res,* 39**,** 20-6.
54. SKURSKA, A., DOLINSKA, E., PIETRUSKA, M., PIETRUSKI, J. K., DYMICKA, V., KEMONA, H., ARWEILER, N. B., MILEWSK, R. & SCULEAN, A. 2015. Effect of nonsurgical periodontal treatment in conjunction with either systemic administration of amoxicillin and metronidazole or additional photodynamic therapy on the concentration of matrix metalloproteinases 8 and 9 in gingival crevicular fluid in patients with aggressive periodontitis. *BMC Oral Health,* 15**,** 63.
55. NARDI, G. M., CESARANO, F., PAPA, G., CHIAVISTELLI, L., ARDAN, R., JEDLINSKI, M., MAZUR, M., GRASSI, R. & GRASSI, F. R. 2020. Evaluation of Salivary Matrix Metalloproteinase (MMP-8) in Periodontal Patients Undergoing Non-Surgical Periodontal Therapy and Mouthwash Based on Ozonated Olive Oil: A Randomized Clinical Trial. *Int J Environ Res Public Health,* 17.
56. LIU, Y., DUAN, D., MA, R., DING, Y., XU, Y., ZHOU, X., ZHAO, L. & XU, X. 2020. The combined use of salivary biomarkers and clinical parameters to predict the outcome of scaling and root planing: A cohort study. *J Clin Periodontol,* 47**,** 1379-1390.
57. BUDUNELI, N., VARDAR, S., ATILLA, G., SORSA, T., LUOTO, H. & BAYLAS, H. 2002. Gingival crevicular fluid matrix metalloproteinase-8 levels following adjunctive use of meloxicam and initial phase of periodontal therapy. *J Periodontol,* 73**,** 103-9.
58. FARHAD, S. Z., AMINZADEH, A., MAFI, M., BAREKATAIN, M., NAGHNEY, M. & GHAFARI, M. R. 2013. The effect of adjunctive low-dose doxycycline and licorice therapy on gingival crevicular fluid matrix metalloproteinase-8 levels in chronic periodontitis. *Dent Res J (Isfahan),* 10**,** 624-9.
59. KURTIS, B., TÜTER, G., SERDAR, M., PINAR, S., DEMIREL, I. & TOYMAN, U. 2007. GCF MMP-8 levels in smokers and non-smokers with chronic periodontitis following scaling and root planing accompanied by systemic use of flurbiprofen. *J Periodontol,* 78**,** 1954-61.
60. CHEN, H. Y., COX, S. W., ELEY, B. M., MÄNTYLÄ, P., RÖNKÄ, H. & SORSA, T. 2000. Matrix metalloproteinase-8 levels and elastase activities in gingival crevicular fluid from chronic adult periodontitis patients. *J Clin Periodontol,* 27**,** 366-9.
61. OZGÖREN, O., DEVELIOGLU, H., GÜNCÜ, G., AKMAN, A. & BERKER, E. 2014. The adjunctive effect of tenoxicam during non-surgical periodontal treatment on clinical parameters and gingival crevicular fluid levels of MMP-8

and TNF-$\alpha$ in patients with chronic periodontitis - randomized, double-blind clinical trial. *Adv Clin Exp Med,* 23**,** 559-65.

62. TÜTER, G., SERDAR, M., KURTIŞ, B., WALKER, S. G., ATAK, A., TOYMAN, U., PINAR, S. & AYKAN, T. 2010. Effects of scaling and root planing and subantimicrobial dose doxycycline on gingival crevicular fluid levels of matrix metalloproteinase-8, -13 and serum levels of HsCRP in patients with chronic periodontitis. *J Periodontol,* 81**,** 1132-9.
63. JENTSCH, H. F., BUCHMANN, A., FRIEDRICH, A. & EICK, S. 2016. Nonsurgical therapy of chronic periodontitis with adjunctive systemic azithromycin or amoxicillin/metronidazole. *Clin Oral Investig,* 20**,** 1765-73.
64. GONÇALVES, P. F., HUANG, H., MCANINLEY, S., ALFANT, B., HARRISON, P., AUKHIL, I., WALKER, C. & SHADDOX, L. M. 2013. Periodontal treatment reduces matrix metalloproteinase levels in localized aggressive periodontitis. *J Periodontol,* 84**,** 1801-8.
65. LEPPILAHTI, J. M., SORSA, T., KALLIO, M. A., TERVAHARTIALA, T., EMINGIL, G., HAN, B. & MÄNTYLÄ, P. 2015. The utility of gingival crevicular fluid matrix metalloproteinase-8 response patterns in prediction of site-level clinical treatment outcome. *J Periodontol,* 86**,** 777-87.
66. SORSA, T., GURSOY, U. K., NWHATOR, S., HERNANDEZ, M., TERVAHARTIALA, T., LEPPILAHTI, J., GURSOY, M., KÖNÖNEN, E., EMINGIL, G., PUSSINEN, P. J. & MÄNTYLÄ, P. 2016. Analysis of matrix metalloproteinases, especially MMP-8, in gingival creviclular fluid, mouthrinse and saliva for monitoring periodontal diseases. *Periodontol 2000,* 70**,** 142-63.
67. DE OLIVEIRA, R. R., SCHWARTZ-FILHO, H. O., NOVAES, A. B., GARLET, G. P., DE SOUZA, R. F., TABA, M., SCOMBATTI DE SOUZA, S. L. & RIBEIRO, F. J. 2009. Antimicrobial photodynamic therapy in the non-surgical treatment of aggressive periodontitis: cytokine profile in gingival crevicular fluid, preliminary results. *J Periodontol,* 80**,** 98-105.
68. HOLMLUND, A., HÄNSTRÖM, L. & LERNER, U. H. 2004. Bone resorbing activity and cytokine levels in gingival crevicular fluid before and after treatment of periodontal disease. *J Clin Periodontol,* 31**,** 475-82.
69. EMINGIL, G., ATILLA, G., SORSA, T., SAVOLAINEN, P. & BAYLAS, H. 2004. Effectiveness of adjunctive low-dose doxycycline therapy on clinical parameters and gingival crevicular fluid laminin-5 gamma2 chain levels in chronic periodontitis. *J Periodontol,* 75**,** 1387-96.
70. REDDY, N. R., ROOPA, D., BABU, D. S., KUMAR, P. M., RAJU, C. M. & KUMAR, N. S. 2012. Estimation of matrix metalloproteinase-3 levels in gingival crevicular fluid in periodontal disease, health and after scaling and root planing. *J Indian Soc Periodontol,* 16**,** 549-52.
71. KOROMANTZOS, P. A., MAKRILAKIS, K., DEREKA, X., OFFENBACHER, S., KATSILAMBROS, N., VROTSOS, I. A. & MADIANOS, P. N. 2012. Effect of non-surgical periodontal therapy on C-reactive protein, oxidative stress, and matrix

metalloproteinase (MMP)-9 and MMP-2 levels in patients with type 2 diabetes: a randomized controlled study. *J Periodontol,* 83**,** 3-10.

72. OKUDA, K., MIYAZAKI, A., MOMOSE, M., MURATA, M., NOMURA, T., KUBOTA, T., WOLFF, L. F. & YOSHIE, H. 2001. Levels of tissue inhibitor of metalloproteinases-1 and matrix metalloproteinases-1 and -8 in gingival crevicular fluid following treatment with enamel matrix derivative (EMDOGAIN). *J Periodontal Res,* 36**,** 309-16.
73. GÓRSKA, R. & NEDZI-GÓRA, M. 2006. The effects of the initial treatment phase and of adjunctive low-dose doxycycline therapy on clinical parameters and MMP-8, MMP-9, and TIMP-1 levels in the saliva and peripheral blood of patients with chronic periodontitis. *Arch Immunol Ther Exp (Warsz),* 54**,** 419-26.
74. AĞAN, S., SÖNMEZ, S. & SERDAR, M. 2006. The effect of topical doxycycline usage on gingival crevicular fluid MMP-8 levels of chronic and aggressive periodontitis patients: a pilot study. *Int J Dent Hyg,* 4**,** 114-21.
75. KURGAN, Ş., FENTOĞLU, Ö., ÖNDER, C., SERDAR, M., ESER, F., TATAKIS, D. N. & GÜNHAN, M. 2016. The effects of periodontal therapy on gingival crevicular fluid matrix metalloproteinase-8, interleukin-6 and prostaglandin E2 levels in patients with rheumatoid arthritis. *J Periodontal Res,* 51**,** 586-95.
76. LEPPILAHTI, J. M., KALLIO, M. A., TERVAHARTIALA, T., SORSA, T. & MÄNTYLÄ, P. 2014. Gingival crevicular fluid matrix metalloproteinase-8 levels predict treatment outcome among smokers with chronic periodontitis. *J Periodontol,* 85**,** 250-60.
